# Investigating disparities in smoking cessation treatment for veterans with multiple sclerosis: A national analysis

**DOI:** 10.1002/brb3.3513

**Published:** 2024-05-02

**Authors:** Carri S. Polick, Paul Dennis, Patrick S. Calhoun, Tiffany J. Braley, Eunice Lee, Sarah Wilson

**Affiliations:** ^1^ Durham VA Health Care System Durham North Carolina USA; ^2^ School of Nursing, Duke University Durham North Carolina USA; ^3^ Department of Psychiatry and Behavioral Sciences Duke University School of Medicine Durham North Carolina USA; ^4^ Department of Neurology Michigan Medicine Ann Arbor Michigan USA; ^5^ Duke University Durham North Carolina USA

**Keywords:** Health equity, Multiple Sclerosis, Smoking cessation, Veterans

## Abstract

**Background and aims:**

Smoking is a risk factor for multiple sclerosis (MS) development, symptom burden, decreased medication efficacy, and increased disease‐related mortality. Veterans with MS (VwMS) smoke at critically high rates; however, treatment rates and possible disparities are unknown. To promote equitable treatment, we aim to investigate smoking cessation prescription practices for VwMS across social determinant factors.

**Methods:**

We extracted data from the national Veterans Health Administration electronic health records between October 1, 2017, and September 30, 2018. To derive marginal estimates of the association of MS with receipt of smoking‐cessation pharmacotherapy, we used propensity score matching through the extreme gradient boosting machine learning model. VwMS who smoke were matched with veterans without MS who smoke on factors including age, race, depression, and healthcare visits. To assess the marginal association of MS with different cessation treatments, we used logistic regression and conducted stratified analyses by sex, race, and ethnicity.

**Results:**

The matched sample achieved a good balance across most covariates, compared to the pre‐match sample. VwMS (*n* = 3320) had decreased odds of receiving prescriptions for nicotine patches ([Odds Ratio]*OR* = 0.86, *p* < .01), non‐patch nicotine replacement therapy (NRT; *OR* = 0.81, *p* < .001), and standard practice dual NRT (*OR* = 0.77, *p* < .01), compared to matches without MS (*n* = 13,280). Men with MS had lower odds of receiving prescriptions for nicotine patches (*OR* = 0.88, *p* = .05), non‐patch NRT (*OR* = 0.77, *p* < .001), and dual NRT (*OR* = 0.72, *p* < .001). Similarly, Black VwMS had lower odds of receiving prescriptions for patches (*OR* = 0.62, *p* < .001), non‐patch NRT (*OR* = 0.75, *p* < .05), and dual NRT (*OR* = 0.52, *p* < .01). The odds of receiving prescriptions for bupropion or varenicline did not differ between VwMS and matches without MS.

**Conclusion:**

VwMS received significantly less smoking cessation treatment, compared to matched controls without MS, showing a critical gap in health services as VwMS are not receiving dual NRT as the standard of care. Prescription rates were especially lower for male and Black VwMS, suggesting that under‐represented demographic groups outside of the white female category, most often considered as the “traditional MS” group, could be under‐treated regarding smoking cessation support. This foundational work will help inform future work to promote equitable treatment and implementation of cessation interventions for people living with MS.

## Graphical abstract

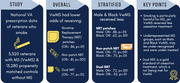

## 1 INTRODUCTION

Multiple sclerosis (MS) is a chronic immune‐mediated disease characterized by demyelination and axonal loss in the central nervous system, contributing to disability and other symptoms (e.g., numbness, tingling, vision issues, fatigue; Shah et al., 2023). MS is the leading cause of non‐traumatic disability in young adults as it is primarily diagnosed between the ages of 20 and 40 (Briggs et al., 2017; Shah et al., 2023). MS has both genetic and environmental risk factors, such as smoking tobacco, which increases the risk of developing MS by 50% (Tanasescu et al., 2018). Smoking is associated with faster disease progression, worse disability, fatigue, depression, and quality of life for people with MS (PwMS; Briggs et al., 2017, 2019; Kahraman et al., 2021; Tanasescu et al., 2018). Smoking tobacco may also hinder the efficacy of disease‐modifying therapies (Bachelet et al., 2016; Hedström et al., 2014) and is associated with higher MS‐related mortality rates (Manouchehrinia et al., 2014). Evidence consistently supports that smoking cessation improves overall and MS‐specific health outcomes (Rodgers et al., 2022; Rosso & Chitnis, 2020).

Prevalence rates of MS have increased over the past 50 years (Wallin et al., 2019). Now, nearly 1 million people in the United States have MS (Multiple Sclerosis International Federation, 2023). Veterans smoke at higher rates, compared to the general US population (Jonk et al., 2005; Wang et al., 2021), which is particularly harmful because smoking increases the risk of MS onset by 40% for women and 80% for men (Hedström et al., 2009). The prevalence of MS among veterans who receive care at the VA nearly doubled from 1999 (141 per 100,000) to 2014 (262 per 100,000; Veterans Affairs, 2021). Although MS is approximately three times more common in women than in men (Wallin et al., 2020), it is notable that the veteran population is disproportionately male. Thus, VA data provide an opportunity to investigate multiple groups under‐represented in MS research, including women veterans and men with MS.

The VA has national policies and programs to reduce smoking and to increase access to evidence‐based treatment for smoking (Hamlett‐Berry, 2004; Smith et al., 2010). Although primary care providers are required to screen for tobacco use annually, they do not usually provide counseling to aid cessation and focus primarily on pharmacological management (Thorndike et al., 2007). Current cessation methods include a combination of counseling and pharmacotherapy, ranging from nicotine replacement therapy (NRT) utilizing patches, gum, and lozenges to prescriptions of bupropion and varenicline. Current cessation medication prescription rates of veterans with MS (VwMS) remain unknown. Prior evidence highlights that only a minor portion of veteran smokers receive smoking cessation pharmacotherapy (Jonk et al., 2005). There is a considerable gap in the literature regarding prescription rates, especially whether prescription practices vary across racial and ethnic groups for VwMS.

The delivery of cessation services should be investigated in tandem with the context of social disparities that affect healthcare. MS is traditionally thought to be far more prevalent in those of Northern European descent. However, recent age‐ and sex‐adjusted evidence revealed that prevalence (per 100,000 people) in Black or African American persons (225.8) was nearly as high as White persons (237.7), which is significantly higher than rates in Hispanic/Latino (69.9) and Asian (22.6) persons (Langer‐Gould et al., 2022). In kind, recent work also suggests healthcare disparities among racial and ethnic minoritized groups with MS. For example, structural barriers that impact healthcare access, like socioenvironmental deprivation, likely underpin findings that Black patients are 28% less likely to have outpatient neurology care (Saadi et al., 2017). In turn, lack of specialty care may lead to missed opportunities to address critical MS‐specific risks such as smoking. Despite recent prioritization of MS health disparities in research, available knowledge on experiences of diverse patient groups remains insufficient (Amezcua et al., 2021). This study aims to evaluate for potential disparities across smoking cessation prescription practices in a national sample of VwMS.

## 2 METHODS

### 2.1 Data source

Data used in the present study are secondary to a primary investigation aimed at identifying sociodemographic predictors of receipt of smoking‐cessation pharmacotherapy among veterans. In this retrospective longitudinal study, national Veterans Health Administration (VHA) electronic health records (EHRs), including outpatient, inpatient, and emergency department care, were used to identify the study cohort of all tobacco users and obtain exposure, outcome, and covariate data (as described below). VHA Health Factor data were used to define smoking status. We followed the Strengthening the Reporting of Observational Studies in Epidemiology reporting guidelines in preparing this article.

### 2.2 Study cohort

The VHA Corporate Data Warehouse (Fihn et al., 2014) was utilized to create a cohort of veterans with and without MS who were identified as tobacco users during a baseline period between October 1, 2017, and September 30, 2018, corresponding to the VA fiscal year 2018. This initial cohort numbered 1,226,697. After dropping 14 tobacco users with out‐of‐range ages (under 18 years or over 105), three with missing data on sex, and 54,802 with missing Area Deprivation Index (ADI) data, the final analytic sample was 1,171,878.

### 2.3 Variable definitions

#### 2.3.1 Exposure

Exposure was defined as having a diagnosis of MS. Consistent with prior VHA EHR research, MS was defined using International Classification of Diseases‐9 (ICD‐9) or ICD‐10. Revision diagnosis codes (see Supporting Information for codes). We required MS documentation at one or more inpatient or two or more outpatient visits with any healthcare service during the baseline period.

#### 2.3.2 Outcomes

Outcomes were receipt of prescriptions for the following smoking‐cessation pharmacotherapy within 12 months of the first health factor indicating positive identification of tobacco use: bupropion, varenicline, non‐patch NRT (e.g., lozenges, gum), patch NRT, and dual‐use NRT (i.e., nicotine patch plus non‐patch NRT).

#### 2.3.3 Demographic and clinical risk factors

Demographic characteristics (age, sex, race/ethnicity, and ADI scores) and clinical risk factors (number of primary care visits, posttraumatic stress disorder [PTSD], depression, non‐PTSD anxiety disorder) were extracted from the EHR and used in propensity‐score matching (PSM). Age, sex, and race/ethnicity (categorized as American Indian or Alaskan Native, Asian, non‐Hispanic Black or African American, Black Hispanic, non‐Black Hispanic, Multiracial, Native Hawaiian or Pacific Islander, non‐Hispanic White) were self‐reported. ADI was measured using an index adapted by Kind and Buckingham (2018) from a measure created by the Health Resources and Services Administration, which provides national percentile rankings (ranging from 1 to 100) of socioenvironmental deprivation at the census block group (i.e., neighborhood) level. ADI are updated at 5‐year intervals; data from 2015 were used for the current study (University of Wisconsin School of Medicine & Public Health, 2022). ADI data were linked to individual veterans using addresses registered during the study period, which are updated quarterly. In the cases of multiple addresses, the address with the lowest corresponding ADI was used. The number of primary care visits during the baseline period was determined by calculating the number of days in which primary care‐related stop codes were recorded in the EHR. Finally, PTSD, depression, and anxiety were identified in a similar manner to MS via ICD codes (see Supporting Information Table S1 for codes). Specifically, we required one or more inpatient or two or more outpatient codes during the study period for positive identification.

### 2.4 PSM data analysis

To derive marginal estimates of the association of MS with receipt of smoking‐cessation pharmacotherapy, we used PSM, which is a quasi‐experimental technique to balance confounding factors of those with and without MS (Blundell & Costa Dias, 2000). Propensity scores were estimated for all 1,171,878 tobacco users in the study cohort using extreme gradient boosting (XGB), a tree‐based machine learning algorithm that iteratively models residuals to improve fit (Chen et al., 2015). Machine learning techniques have been demonstrated to produce less biased estimates than standard parametric techniques because they are less prone to misspecification (e.g., overfitting, assumptions of linearity; Lee et al., 2010; Westreich et al., 2010). To deter under‐ and overfitting of the propensity model, we randomly partitioned the sample into training and test sets along a 70/30 split. The training set was used to develop and tune the model, which included as predictors the aforementioned demographic and clinical factors. The test set was used to evaluate the models’ performance on heretofore unseen data. Due to the low frequency of MS (*n *= 3320, 0.3%), we weighted MS cases by their inverse probability. Ten‐fold cross‐validation was applied to the training set to derive optimal values of the XGB hyperparameters corresponding to eta, maximum tree depth, ratio of predictors subsampled, percentage of observations subsampled, and boosting iterations. Hyperparameters were optimized via grid search on the accuracy of predictions captured as the area under the receiver‐operator curve (AUROC). Once the optimal model hyperparameters were determined through the cross‐validation process, the model was applied to the full training and test sets, and AUROC corresponding to agreement between observed and predicted values were calculated for each, with values close to 0.50 reflecting little to no agreement and values close to 1 reflecting near perfect agreement. As a final step, the XGB model was used to generate propensity scores representing predicted probabilities of PTSD status for the entire matched sample.

Nearest neighbor matching was performed on the XGB propensity scores at a 4:1 ratio of tobacco users without MS to those with MS, using a caliper of 0.20 and restricting matches to tobacco users with no more than a 2‐year age difference. Standardized mean differences were used to confirm covariate balance among patients with MS and matched controls. To assess the marginal association of MS with each of the aforementioned pharmacotherapies, we used logistic regression. As a final step, we conducted stratified analyses, stratifying by sex and race/ethnicity, categorized as Black/African American, White, or other/missing due to small cell sizes.

Analyses were conducted using R, version 4.1.2. XGB and k‐fold cross‐validation was conducted using the “caret” package for R, version 6.0‐94. Nearest neighbor PSM was conducted using the “MatchIt” package, version 4.5.3. Two‐sided *p* < .05 indicated statistical significance.

## 3 RESULTS

Summary statistics and MSDs for the pre‐match and matched samples are displayed in Table 1. In contrast to the pre‐match sample, the matched sample achieved good balance across most covariates, with no MSDs > 0.20. However, MSDs for number of primary care visits, PTSD, and two race/ethnicity categories (White and Black/African American) exceeded 0.10; thus, these variables were covaried in the logistic models. The final XGB model used to produce propensity scores for matching demonstrated modest fit on both the training (AUROC = 0.68) and test sets (AUROC = 0.66), indicating that the selected covariates were not strong predictors of MS. Sex, age, depression, ADI, and number of primary care visits were the most important features in the XGB model with regard to their involvement in splits relatively early on in the propagation of trees (see Supporting Information Figure S1).

**TABLE 1:** Demographic and clinical characteristics of pre‐match and matched samples.

	Pre‐match sample			Matched sample		
	No MS	MS		No MS	MS	
	(*n* = 1,168,558)	(*n* = 3320)	SMD	(*n* = 13,280)	(*n* = 3320)	SMD
Age (years) M(SD)	57.57 (14.66)	55.16 (11.99)	−0.180	55.18 (11.96)	55.16 (11.99)	−0.001
Area Deprivation Index, national rank M(SD)	61.43 (24.64)	57.73 (24.43)	−0.151	57.91 (24.85)	57.73 (24.43)	−0.007
Primary care visits in previous year M(SD)	5.73 (15.31)	6.10 (14.25)	0.025	8.14 (17.85)	6.10 (14.25)	−0.126
Sex (female) *n*(%)	87,912 (7.5%)	715 (21.5%)	0.406	2860 (21.5%)	715 (21.5%)	0.000
Race/Ethnicity *n*(%)						
American Indian/Native Alaskan	9162 (0.8%)	18 (0.5%)	−0.030	119 (0.9%)	18 (0.5%)	−0.042
Asian	7634 (0.7%)	7 (0.2%)	−0.067	103 (0.8%)	7 (0.2%)	−0.081
Black/African American	232,675 (19.9%)	598 (18.0%)	−0.048	2945 (22.2%)	598 (18.0%)	−0.104
Hispanic, Black	3355 (0.3%)	13 (0.4%)	0.018	45 (0.3%)	13 (0.4%)	0.009
Hispanic, not Black	58,238 (5.0%)	131 (3.9%)	−0.050	636 (4.8%)	131 (3.9%)	−0.041
Multiracial	9847 (0.8%)	22 (0.7%)	−0.021	121 (0.9%)	22 (0.7%)	−0.028
Native Hawaiian/Pacific Islander	7447 (0.6%)	14 (0.4%)	−0.030	84 (0.6%)	14 (0.4%)	−0.029
White	787,334 (67.4%)	2381 (71.7%)	0.094	8621 (64.9%)	2381 (71.7%)	0.147
Missing	52,866 (4.5%)	136 (4.1%)	−0.021	606 (4.6%)	136 (4.1%)	−0.023
PTSD *n*(%)	212,724 (18.2%)	576 (17.3%)	−0.022	3215 (24.2%)	576 (17.3%)	−0.170
Depression *n*(%)	219,354 (18.8%)	1089 (32.8%)	0.325	4338 (32.7%)	1089 (32.8%)	0.003
Anxiety *n*(%)	118,245 (10.1%)	433 (13.0%)	0.091	1947 (14.7%)	433 (13.0%)	−0.047

Abbreviations: MS, multiple sclerosis; PTSD, posttraumatic stress disorder; SMD, standardized mean difference.

### 3.1 MS and tobacco‐cessation pharmacotherapy prescriptions

According to results of the logistic models, a diagnosis of MS was associated with decreased odds of receiving prescriptions of non‐patch NRT ([Odds Ratio]*OR* = 0.81, *p* < .001), nicotine patches (*OR* = 0.86, *p* = .008), and dual NRT (*OR* = 0.77, *p* = .004; see Table 2). Tobacco users with MS did not have increased or decreased odds of receiving prescriptions for bupropion (*OR *= 0.94, *p *= .62) or varenicline (*OR *= 1.01, *p* = .92). Men with MS had lower odds of receiving prescriptions for non‐patch NRT (*OR *= 0.77, *p* < .001) and dual NRT (*OR* = 0.72, *p* = .001), compared to men without MS. Both men (*OR* = 0.88, *p* = .05) and women (*OR *= 0.81, *p* = .06) with MS had marginally lower odds of receiving prescriptions for nicotine patches compared to non‐MS groups. Black (*OR *= 0.75, *p* = .042) and White tobacco users with MS (*OR *= 0.84, *p* = .017) had lower odds of receiving prescriptions for non‐patch NRT than their counterparts without MS. However, across racial and ethnic groups, only Black tobacco users with MS had lower odds of receiving prescriptions for nicotine patches (*OR* = 0.62, *p* < .001) and dual NRT (*OR *= 0.52, *p* = .004), compared to their counterparts without MS.

**TABLE 2:** Logistic models of smoking‐cessation pharmacotherapy prescriptions received in MS cohort compared to non‐MS cohort.

	NRT (patch)	NRT (non‐patch)	Dual NRT	Bupropion	Varenicline
**Full matched MS sample**					
OR (95% CI)	0.86 (0.78–0.96)	0.81 (0.72–0.92)	0.77 (0.65–0.92)	0.94 (0.74–1.19)	1.01 (0.84–1.21)
*p*‐value	**.008**	**<.001**	**.004**	.62	.92
**Sex strata**					
Male					
OR (95% CI)	0.88 (0.78–1.00)	0.77 (0.67–0.88)	0.72 (0.59–0.88)	0.94 (0.71–1.24)	1.05 (0.85–1.29)
*p*‐value	**.05**	**<.001**	**.001**	.67	.67
Female					
OR (95% CI)	0.81 (0.65–1.01)	0.97 (0.76–1.23)	0.96 (0.69–1.34)	0.93 (0.59–1.47)	0.91 (0.64–1.30)
*p*‐value	.06	.81	.82	.76	.61
**Race/ethnicity strata**					
Black/African American					
OR (95% CI)	0.62 (0.48–0.80)	0.75 (0.57–0.99)	0.52 (0.34–0.81)	0.89 (0.52–1.52)	0.76 (0.44–1.32)
*p*‐value	**<.001**	**.042**	**.004**	.66	.33
White					
OR (95% CI)	0.93 (0.82–1.05)	0.84 (0.73–0.97)	0.83 (0.67–1.01)	1.00 (0.75–1.32)	1.04 (0.85–1.27)
*p*‐value	.24	.017	.06	.99	.7
Other/missing					
OR (95% CI)	1.02 (0.73–1.43)	0.76 (0.52–1.13)	0.96 (0.57–1.62)	0.68 (0.30–1.50)	1.10 (0.57–2.15)
*p*‐value	.89	.17	.89	.34	.77

*Note*: Models for the full matched sample and sex strata included as covariates number of primary care visits, posttraumatic stress disorder (PTSD), and race/ethnicity. Race/ethnicity‐stratified models included as covariates number of primary care visits and PTSD.

Abbreviations: MS, multiple sclerosis; NRT, nicotine replacement therapy; OR, Odds Ratio; CI, Confidence Interval.

Further, post hoc Chi‐squared and *t*‐tests were used to compare the receipt of any cessation support (i.e., NRT, bupropion, and/or varenicline) versus no support, across key demographic and clinical characteristics within the MS subgroup. VwMS who received any prescription were significantly younger, had higher ADI, more primary care visits, and higher prevalence rates of anxiety, depression and PTSD, all at the *p* < .001 level. There was a significantly lower proportion of males in the group that received any prescription (75.9% vs, 79.3%; Supporting Information Table S2).

## 4 DISCUSSION

This study is the first to evaluate smoking cessation prescription likelihood for PwMS and uncovers disparities in prescription practices. VwMS received significantly less smoking cessation support across multiple modalities, when compared to matched controls without MS. Despite smoking being especially detrimental to MS disability and disease course, there was not a significant difference in bupropion or varenicline prescription rates between VwMS and the controls without MS. This may highlight a potential lack of focused effort to specifically reduce smoking in VwMS. However, the lack of difference may just be the result of similarly low rates of bupropion and varenicline prescriptions across both the MS cohort and the case‐matched controls, as these all ranged from only 3% to 8%.

Treatment barriers may include determining which healthcare service provider is primarily responsible for cessation management for VwMS, and difficulties coordinating between services as some MS patients may only seek care from neurology providers. For example, primary care providers may complete the required smoking status screening, yet may hesitate to prescribe cessation aids due to concerns of exacerbating neurological issues or potential medication interactions (Polick et al., 2023). In the nascent research in this area, there is recent evidence that most PwMS who smoke find it highly important to receive cessation information from someone knowledgeable about MS (Weld‐Blundell et al., 2022). However, some neurology providers feel that they should not manage cessation treatment, in part, due to less frequent contact (e.g., one to two times a year) with the patient, compared to primary care (Grech et al., 2021). As the complexity of the MS treatment landscape heightens, neurology providers have to prioritize fitting a neuro exam, reviewing symptoms, ongoing brain imaging, MS medications, and patient questions within a 30‐min appointment. Thus, cessation management may not be feasible through the current neurology practice structure. While the majority of smokers report interest in quitting, relatively few are referred to specialty smoking cessation clinics; and many who attend specialty clinics seem to drop out prematurely. Additionally, current specialty cessation services would not provide MS‐focused care, a priority for PwMS (Hunter et al., 2021). Bolstering neurology‐focused case‐management services or transmural models of care, where a clinician (e.g., MS nurse, physician's assistant, physician consult) is an integrated liaison between specialty care and primary care, could help address these critical gaps between fragmented services.

Prescription rates also varied by personal demographic factors. Male VwMS had significantly lower odds of being prescribed nicotine patches, non‐patch NRT, and dual NRT, compared to matched controls without MS. Black VwMS also had significantly lower odds of being prescribed nicotine patches, non‐patch NRT, and dual NRT than matched controls. Taken together, this suggests that under‐represented demographic groups outside of the White female category, most often considered as the “traditional MS” group, could be under‐treated regarding smoking cessation support. Further, given that dual NRT is among the gold standards of care, this work highlights that cessation support for all VwMS, especially for males (when stratified by sex) and Black VwMS (when stratified by race and ethnicity), falls short. While we cannot determine whether the disparity stems from individual patient factors, inter‐personal factors with health providers, or health system factors, social determinants of health lens posits that there are likely barriers across multiple levels, especially as this trend is seen across other access and prescription practices. In a review of care access inequalities in MS, males and Black PwMS were less likely to see a neurologist, men were less likely to access lifestyle wellness services or receive relapse and bladder medications, and Black PwMS were less likely to receive prescription for depression and anxiety (Buchanan et al., 2004; Marrie et al., 2007; Minden et al., 2008; Plow et al., 2010; Roddam et al., 2019; Windt et al., 2013). It is critically important to continue raising awareness of the MS burden among diverse populations to combat potential bias of healthcare providers and find ways to promote equitable treatment in the context of MS and overall.

### 4.1 Strengths

This study has many strengths including a large sample size, with a large number of males and Black individuals with MS, which are usually underrepresented in MS research. The results are generalizable to all VwMS who smoke and receive care at the VA. The case‐matching design provides particularly robust results that are less vulnerable to bias.

### 4.2 Limitations

Limitations include the inability to determine the prescriber service (e.g., neurology, primary care, specialty smoking cessation) or primary intent of a bupropion prescription (e.g., smoking cessation or depression). However, controls were matched across mental health conditions. Results may not be representative of VwMS who get their care outside of the VHA.

## 5 CONCLUSION

This study revealed disparate rates of smoking cessation treatment for all VwMS, compared to matched controls without MS, especially for male and Black VwMS. This work lays the foundation for future work to investigate and address smoking cessation treatment services and disparities among VwMS. Given the inability to determine which healthcare service provided treatment and whether VwMS engaged with referrals to smoking cessation clinics, the treatment pathway following a positive smoking screening should be investigated. These pathways, barriers, and facilitators to accessing and receiving equitable cessation support should eventually inform hospital policies and programs. Further, these factors could be developed into a targeted cessation intervention for PwMS, as no such intervention exists.

## AUTHOR CONTRIBUTIONS

**Carri S. Polick**: Conceptualization; writing—original draft; writing—review and editing. **Paul Dennis**: Conceptualization; formal analysis; writing—original draft; writing—review and editing. **Patrick S. Calhoun**: Conceptualization; writing—review and editing; supervision. **Tiffany J. Braley**: Writing—original draft; writing—review and editing; supervision. **Eunice Lee**: Writing—review and editing; writing—original draft. **Sarah Wilson**: Writing—review and editing; conceptualization; funding acquisition; supervision.

## CONFLICT OF INTEREST STATEMENT

The authors declare there no conflicts of interest.

### PEER REVIEW

The peer review history for this article is available at https://publons.com/publon/10.1002/brb3.3513.

## Supporting information

TABLE S1 ICD codes for data pull.
FIGURE S1 Variable importance from XGBoost propensity model of MS.

TABLE S2 Bivariate analyses comparing cessation prescription receipt across MS subgroup characteristics.

## Data Availability

A deidentified dataset will be made available upon request.
