# Supplementary material for: Investigating disparities in smoking cessation treatment for veterans with multiple sclerosis: A national analysis
Source: Brain Behav. 2024 May 2;14(5):e3513. doi: 10.1002/brb3.3513 (PMC11066415; doi:10.1002/brb3.3513)

**Supplementary Table 1. ICD Codes for data pull**

| MS | ICD-9 | 340.X |
| --- | --- | --- |
|  | ICD-10 | G35.X |
| PTSD | ICD-9 | 309.81 |
|  | ICD-10 | F43.1, F43.10, F43.11, F43.12 |
| Depression | ICD-9 | 296.2, 296.21, 296.22, 296.23, 296.24, 296.25, 296.26, 296.3, 296.31, 296.32, 296.33, 296.34, 296.35, 296.36 |
|  | ICD-10 | F32.0, F32.1, F32.2, F32.3, F32.4, F32.5, F32.9, F33.0, F33.1, F33.2, F33.3, F33.40, F33.41, F33.42, F33.8, F33.9 |
| Anxiety | ICD-9 | 300.01, 300.02, 300.09 |
|  | ICD-10 | F41.0, F41.1, F41.3, F41.8, F41.9 |

**Supplementary Figure 1. Variable importance from XGBoost propensity model of MS**


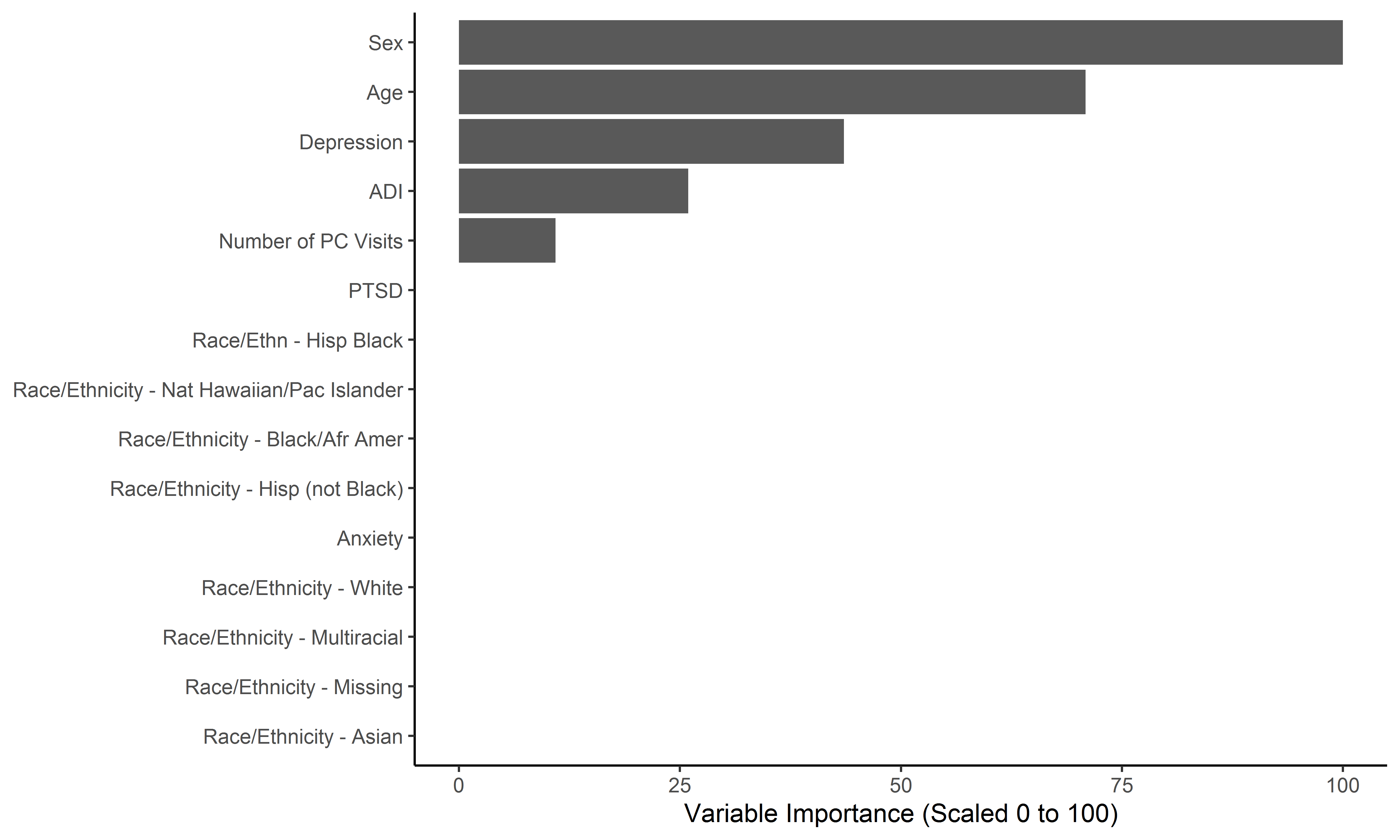

Supplement: Supplementary file 1 — TABLE S1 ICD codes for data pull. FIGURE S1 Variable importance from XGBoost propensity model of MS. [file BRB3-14-e3513-s001.docx]
