# Supplementary material for: Investigating disparities in smoking cessation treatment for veterans with multiple sclerosis: A national analysis
Source: Brain Behav. 2024 May 2;14(5):e3513. doi: 10.1002/brb3.3513 (PMC11066415; doi:10.1002/brb3.3513)
Supplement: Supplementary file 2 — TABLE S2 Bivariate analyses comparing cessation prescription receipt across MS subgroup characteristics. [file BRB3-14-e3513-s002.docx]

**Supplementary Table 2.** Bivariate analyses comparing cessation prescription receipt across MS subgroup characteristics

|  | No Rx | Any Rx | p-value |
| --- | --- | --- | --- |
|  | (n = 2490) | (n = 830) |  |
| Age (years) M(SD) | 55.65 (12.13) | 53.68 (11.42) | <0.001 |
| Area Deprivation Index, national rank M(SD) | 56.82 (24.58) | 60.43 (23.82) | <0.001 |
| Primary care visits in previous year M(SD) | 5.22 (12.54) | 8.75 (18.19) | <0.001 |
| Sex (Male) n(%) | 1975 (79.3) | 630 (75.9) | 0.043 |
| Race/Ethnicity n(%) |  |  | 0.529 |
| American Indian/Native Alaskan | 12 ( 0.5) | 6 ( 0.7) |  |
| Asian | 6 ( 0.2) | 1 ( 0.1) |  |
| Black/African American | 455 (18.3) | 143 (17.2) |  |
| Hispanic, Black | 10 ( 0.4) | 3 ( 0.4) |  |
| Hispanic, not Black | 108 ( 4.3) | 23 ( 2.8) |  |
| Missing | 105 ( 4.2) | 31 ( 3.7) |  |
| Multiracial | 15 ( 0.6) | 7 ( 0.8) |  |
| Native Hawaiian/Pacific Islander | 11 ( 0.4) | 3 ( 0.4) |  |
| White | 1768 (71.0) | 613 (73.9) |  |
| PTSD n(%) | 385 (15.5) | 191 (23.0) | <0.001 |
| Depression n(%) | 748 (30.0) | 341 (41.1) | <0.001 |
| Anxiety n(%) | 289 (11.6) | 144 (17.3) | <0.001 |

*Chi-Squared and T-tests used as appropriate.
